# Influence of the COVID-19 Pandemic on Mood and Training in Australian Community Tennis Players

**DOI:** 10.3389/fspor.2021.589617

**Published:** 2021-03-18

**Authors:** Mitchell Turner, Philipp Beranek, Shane L. Rogers, Kazunori Nosaka, Olivier Girard, Travis Cruickshank

**Affiliations:** ^1^School of Medical and Health Sciences, Edith Cowan University, Joondalup, WA, Australia; ^2^Exercise Medicine Research Institute, Edith Cowan University, Joondalup, WA, Australia; ^3^School of Arts and Humanities, Edith Cowan University, Perth, WA, Australia; ^4^Centre for Exercise and Sports Science Research, Edith Cowan University, Joondalup, WA, Australia; ^5^School of Human Sciences (Exercise and Sport Science), The University of Western Australia, Perth, WA, Australia; ^6^Perron Institute for Neurological and Translational Science, Perth, WA, Australia

**Keywords:** mental health, mood, training, tennis, community

## Abstract

The COVID-19 outbreak has led to the implementation of strict restrictions in Australia, which have severely impacted sporting activities. Tennis is played by 6.2% of the population within the Oceania region, and is a valuable sport for maintaining social, mental, and physical health. Current literature indicates the COVID-19 pandemic has negatively affected the mental health of Australian residents. The aim of this study was to investigate changes in training and match play due to the outbreak, and its effects on emotional well-being of Australian senior tennis players. Additionally, explore any differences between middle aged (41–60 years) and senior (61 years and over) Australian tennis players. An online survey was used to assess training and match play habits, as well as ascertain the emotional well-being of tennis players. The survey was active from 24th April 2020 until 6th June 2020. Participants were adult (41+ years) tennis players from Australia. Wilcoxon signed rank tests were performed to check for differences in training hours and tennis matches played. Mann–Whitney *U* tests were used to assess the difference in Brief Emotional Experience Scale (BEES) scores, employment status as well as the training hours and tennis matches played between the two age groups. A Kendall's Tau B correlation test was performed to assess the associations of training, match play and demographic characteristics with BEES scores. Kruskal–Wallis tests assessed differences in BEES scores between participants of differing match play formats, tennis experience and cessation of tennis training time periods. There were 245 respondents who met our inclusion criteria. Tennis training hours along with the tennis matches played significantly decreased during COVID-19 compared to pre-COVID-19, 85.09 and 88.48%, respectively. No significant (*p* > 0.05) differences were observed between age groups for any of the training modality hours, nor was there any significant difference in number of tennis matches played. The participants average BEES score was 0.99 ± 1.27, indicating that respondents had a positive emotional well-being during the COVID-19 pandemic. The emotional well-being of the senior group was significantly (*P* = 0.002) higher than the middle aged group. Together, our results show that training and tennis match play decreased during the COVID-19 pandemic, however the emotional well-being of senior tennis players in Australia appeared to not be negatively affected.

## Introduction

The pervasive and deadly nature of severe acute respiratory syndrome coronavirus 2 (COVID-19) has led to implementation of strict restrictions in Australia. On the 20th of March 2020, the Australian government began executing restriction policies, part of which resulted in the closure of all gymnasiums and sporting facilities as well as state borders by the end of March 2020 (Government of Austrlia, 2020). These restrictions severely impacted sporting activities at elite and community levels. The aforementioned restrictions are expected to have a severe impact on the physical, psychological, and social health of athletes (Eime et al., 2013). This is of concern as sport is a fundamental part of Australian culture, with an estimated 8.4 million adults participating in sporting activities every year (Hughes et al., 2020).

Existing literature shows that COVID-19 has negatively impacted on the mental health of Australians, with studies noting significantly elevated stress, anxiety and depressive symptomology (Newby et al., 2019; Fisher et al., 2020). Whilst informative, these studies have cast a wide net with participants including anyone in Australia of at least 18 years of age. Additionally, physical activity was only measured by Newby, O'Moore (Newby et al., 2019) who found less than half of the respondents met the Australian recommendations of at least 150 min of moderate physical activity per week. To date, no studies have investigated if these negative effects were present in physically active individuals especially seniors who had been regularly participated in community sport, such as tennis before the COVID-19 pandemic.

Tennis is one of the most popular sports in the world, with 1.12% of the world's population participating in tennis. Within Oceania (geographical region that includes Australia) an estimated 6.2% of the population participate in tennis, with a relatively equal distribution of females (47%) and males (Federation IT, 2019). The vast majority individuals engaging in tennis participate at a community level and contrary to many other sports, tennis is played across the entirety of the age spectrum and is therefore considered a valuable sport for maintaining healthy physical activity levels in middle aged and older adults. Individuals who participate in tennis training and match play at a community level, therefore provide an important sample to assess the impact of the COVID-19 pandemic, including the subsequent government restrictions.

Therefore, the aim of this study was twofold; (1) to assess changes in training hours and matches played per week due to the COVID-19 pandemic, and (2) to investigate whether changes in these and other factors (gender, age, income, tennis experience, match play format, or period since training cessation), as a result of the COVID-19 pandemic, influenced the emotional well-being of Australian senior tennis players.

## Methods

### Study Design

A cross-sectional study was utilized to investigate the impact of the COVID-19 pandemic on senior tennis players across Australia. An online survey (Supplementary Material A) was developed to assess changes in training and match play habits, as well as ascertain the emotional well-being of tennis players. The survey was active from 24th April 2020 until 6th June 2020. During this time, the Australian government enforced its strictest restrictions. This study was reviewed and approved by Edith Cowan University Human Research Ethics Committee (2020-01367).

### Participants

Participants included adult (41+ years of age) tennis players from tennis clubs across Australia. Participants were provided with a link to the survey via social media platforms, their local tennis club or state tennis organization. An information sheet was displayed at the start of the survey and available for download. Participants had to signify consent by ticking a box before continuing on to the survey. General demographic information was first collected, including participants' age range, gender, relationship status, employment, income status, and tennis experience, as well as their thoughts on the COVID-19 pandemic and how it has been handled by their tennis organization.

### Measures

#### Training and Match Play

Training was evaluated using survey questions focused on training modality, hours, location, and equipment. Tennis match play was evaluated using questions querying the number of matches participants played each week. Participants had to report how many hours per week they played tennis and how many tennis matches on average they played per week prior to the COVID-19 pandemic. In order to evaluate a potential change due to the COVID-19 pandemic, the same questions were repeated but in regard to the time point at which they were completing the survey. Because changes in their tennis training and match play volume due to the COVID-19 pandemic could be compensated for by changes in other activities, additional questions focussing on participants' training activities were also included. Participants had to provide information about their weekly training volume of endurance, strength, speed and agility, flexibility, and mobility, coordination (e.g., balance), as well as mental or tactic training prior to the COVID-19 pandemic and at the time point at which they were completing the survey. In order to get a better picture of their training routine and opportunities during COVID-19 pandemic, participants had to report where they were performing the off-court training and what equipment they had access to. For participants who had ceased their tennis training, the time point and reason for this were requested. Those participants who did not perform any form of off-court training during the COVID-19 pandemic were also requested to provide a reason in order to better understand their mindset and motivation.

#### Emotional Well-Being

The Brief Emotional Experience Scale (BEES) is a self-report mood measure, which indicates a participants level of emotional distress (Rogers et al., 2016). It contains positive and negative adjectives, which form complementary pairs covering depression (happy, sad), anxiety (afraid, confident), and stress (worried, calm). Participants were required to rate these adjectives on a response scale (not at all, a little bit, quite a bit, a lot, extremely) according to their feelings over the past month. The average score of the negative adjectives has then been subtracted from the average score of the positive adjectives. This resulted in an overall score which indicates the participant's mood state. This score can range from −3 to +3, representing a greater negative emotion or greater positive emotion, respectively. The BEES has been previously used with an adult general population (Rogers et al., 2016), and has good convergent validity with several established questionnaires (Rogers et al., 2016; Skead et al., 2018).

### Statistical Analysis

Participants were stratified into two age categories, middle aged (41–60 years) or senior (61+ years; Liu et al., 2020). The percentage of responses per group were displayed for demographic and training information. The mean and standard deviation of each group was reported for training hours and matches played. The data was checked for normality using a Shapiro-Wilk test, confirming that data was not normally distributed. Wilcoxon signed rank test was performed to check for differences in training hours and tennis matches played per week between pre- and during COVID-19 periods. A Mann–Whitney *U* test was performed to assess the difference in BEES scores, employment status as well as the training hours and tennis matches played per week between the two age groups. A Kendall's Tau B correlation test was then performed to evaluate potential associations between training hours and tennis matches played pre- and during COVID-19 and the BEES. Gender, age range, and loss of income were included as covariates in the aforementioned analysis. Kruskal-Wallis tests were performed to determine if the emotional well-being differed in participants as a result of their match play format (singles, doubles, or both), tennis experience or for the time since tennis training ceased (for those who stopped training). Holm-Bonferroni corrections were applied to all *p*-values.

## Results

### Participants

**Figure 1:**
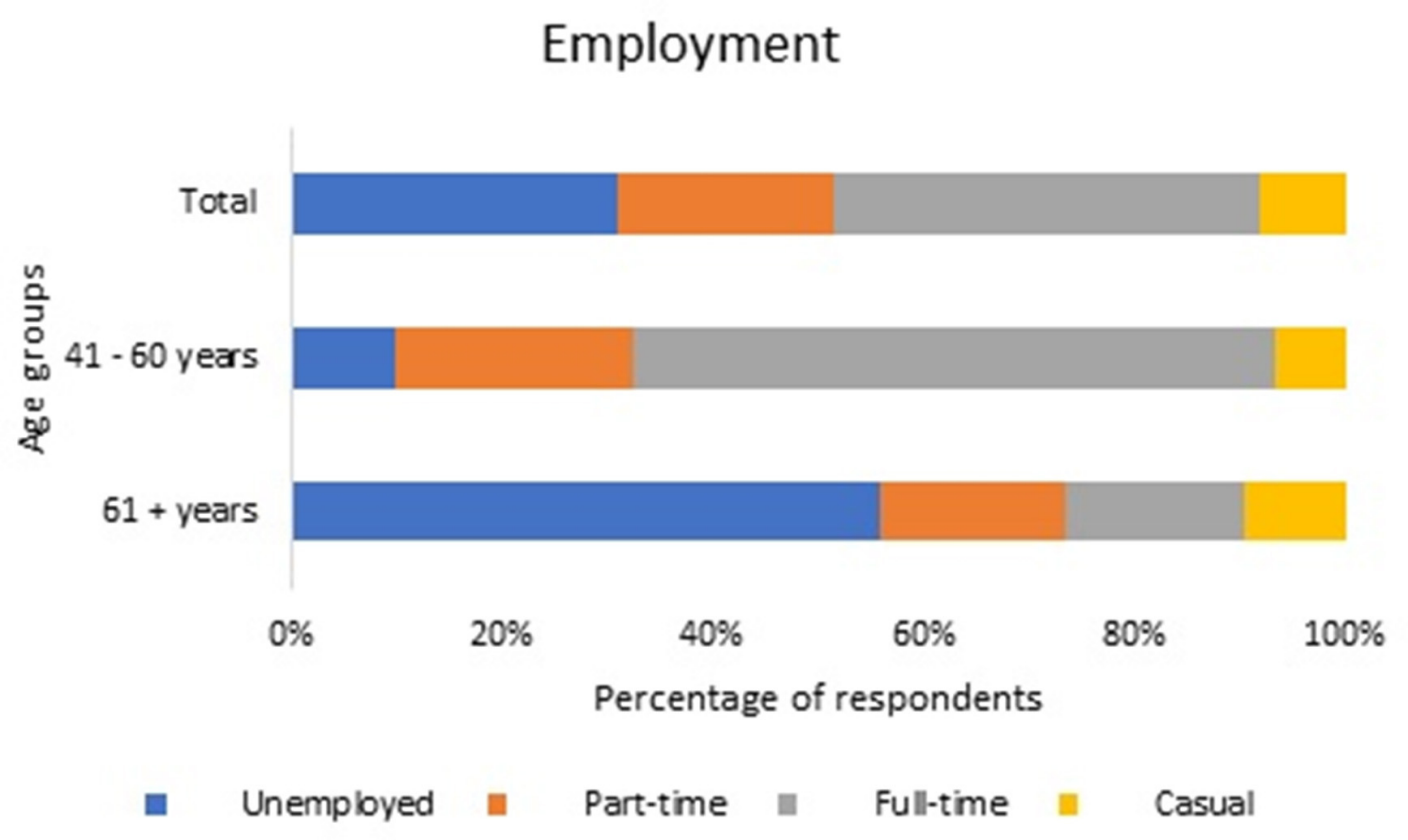
The percentage of respondents who were either unemployed, part-time, full-time, or casually employed prior to the COVID-19 pandemic.

There were 245 respondents who met our inclusion criteria, which was a regular tennis player of 41+ years of age in Australia. This included 132 middle aged (41–60 years) participants as well as 113 senior (61+ years) participants. Of the participants who completed the survey, 47% were female which represents the same distribution of females playing tennis internationally (ITF global tennis report 2019), and 82.5% of participants were in a committed relationship and 84.5% had been playing tennis for over 20 years. None of the participants reported testing positive to COVID-19 and 76.5% of participants thought the response of their tennis organization to the COVID-19 pandemic was either good or very good (Table 1). Employment status prior to the COVID-19 pandemic significantly differed (*p* < 0.001) between the two age groups with 60.6% of participants in the middle-aged group working full-time, compared to 16.8% in the senior group (Figure 1).

**Table 1:** Participant information displayed as percentage of responses.

**Participant demographics**			
**Parameters**	**Total** **(*****n*** **= 245)**	**41–60 years** **(*****n*** **= 132)**	**61 + years** **(*****n*** **= 113)**
**Age range (years)**			
41**–**50	20.8	38.6	**–**
51**–**60	33.1	61.4	**–**
61**–**70	32.2	**–**	69.9
71 +	13.9	**–**	30.1
**Sex**			
Male	51.8	52.3	51.3
Female	47.8	47.0	48.7
Do not want to identify	0.4	0.8	0.0
**Relationship status**			
Single	15.5	11.4	20.4
In a relationship	82.5	87.1	77.0
Other	2.0	1.5	2.7
**Income**			
Loss of income	34.3	35.6	32.7
No loss of income	65.7	64.4	67.3
**Tennis experience (years)**			
1**–**3	1.2	1.5	0.9
3**–**5	2.0	3.0	0.9
5**–**10	2.9	5.3	0.0
10**–**15	4.9	6.8	2.7
15**–**20	4.5	5.3	3.5
20 +	84.5	78.0	92.0
**Have you contracted COVID-19**			
Yes	0.0	0.0	0.0
No	95.1	91.7	99.1
Unsure	4.9	8.3	0.9
**Response of tennis organization**			
Very good	39.8	36.4	42.5
Good	36.7	34.9	38.9
Uncertain	12.2	14.4	9.7
Poor	7.4	8.3	6.2
Very poor	4.5	6.1	2.7

### On/Off Court Training and Tennis Match Play

The tennis training hours along with the tennis matches played per week significantly decreased when compared to pre COVID-19, 85.1 and 88.5%, respectively (Table 2). Interestingly, endurance as well as flexibility and mobility training hours per week did not differ significantly (*p* = 0.900 and *p* = 0.730, respectively). No significant (*p* > 0.05) differences were observed between age groups for any of the training modality hours, nor was there any significant difference in tennis match play per week.

**Table 2:** Training hours per week for various modalities and tennis matches played per week, before (pre-) and during COVID-19 presented as mean ± standard deviation.

**Training hours and matches played**			
**Totals (*****n*** **= 245)**	**Pre-COVID-19**	**During COVID-19**	***P*****-value** **(corrected)**
**Training (hours per week)**			
Tennis	3.89 ± 3.01	0.58 ± 1.40	<0.001^*^
Strength	1.27 ± 2.05	0.83 ± 1.57	<0.001^*^
Speed and agility	0.77 ± 1.82	0.49 ± 1.36	0.002^*^
Endurance	1.47 ± 2.36	1.43 ± 2.31	0.900
Flexibility and mobility	1.28 ± 2.15	1.13 ± 1.79	0.730
Coordination	0.89 ± 2.27	0.62 ± 1.63	0.004^*^
Mental/tactics	0.69 ± 2.19	0.52 ± 1.73	0.224
**Tennis matches**	2.43 ± 1.56	0.28 ± 0.85	<0.001^*^
**41–60 years (*****n*** **= 132)**	**Pre COVID-19**	**During COVID-19**	***P*****-value** **(corrected)**
**Training (hours per week)**			
Tennis	3.88 ± 3.21	0.51 ± 1.33	<0.001^*^
Strength	1.15 ± 1.79	0.80 ± 1.50	0.008^*^
Speed and agility	0.55 ± 1.15	0.41 ± 1.01	0.207
Endurance	1.27 ± 1.92	1.50 ± 2.37	0.469
Flexibility and mobility	1.05 ± 1.43	1.19 ± 1.86	0.517
Coordination	0.61 ± 1.42	0.49 ± 1.29	0.362
Mental/tactics	0.37 ± 1.18	0.39 ± 1.15	0.968
**Tennis matches**	2.37 ± 1.37	0.20 ± 0.75	<0.001^*^
**61 + years (*****n*** **= 113)**	**Pre COVID-19**	**During COVID-19**	***P*****-value** **(corrected)**
**Training (hours per week)**			
Tennis	3.89 ± 2.76	0.66 ± 1.48	<0.001^*^
Strength	1.40 ± 2.30	0.87 ± 1.64	0.003^*^
Speed and agility	1.03 ± 2.35	0.59 ± 1.68	0.022^*^
Endurance	1.69 ± 2.77	1.35 ± 2.23	0.111
Flexibility and mobility	1.55 ± 2.74	1.05 ± 1.72	0.034^*^
Coordination	1.22 ± 2.94	0.76 ± 1.95	0.021^*^
Mental/tactics	1.05 ± 2.92	0.68 ± 2.21	0.034^*^
**Tennis matches**	2.50 ± 1.76	0.37 ± 0.94	<0.001^*^

*No significant differences between age groups were observed for any training modality hours or tennis match play per week*.

* *Significant after corrections*.

The majority of participants (76.3%) played only doubles tennis matches as opposed to singles and singles and doubles matches. 70.2% of respondents attributed the inability to perform tennis training during the COVID-19 pandemic to restrictions to tennis courts. Nearly half (45.3%) of the participants were undertaking some form of exercise training at home during the COVID-19 pandemic. Free weight and bands/tubes represented the most common pieces of equipment used by participants for training (Table 3).

**Table 3:** The percentage of responses regarding the training and tennis match play of participants during the COVID-19 pandemic.

**Parameters**	**Total** **(*n* = 245)**	**41–60 years** **(*n* = 132)**	**61 + years** **(*n* = 113)**
**Format**			
Singles	3.7	5.3	1.8
Doubles	76.3	65.9	88.5
Singles and doubles	20.0	28.8	9.7
**When tennis training ceased**	**(*****n*** **= 187)**	**(*****n*** **= 104)**	**(*****n*** **= 83)**
3–4 weeks ago	6.4	4.8	8.4
1–2 months ago	69.0	74.0	62.7
3–4 months ago	20.9	18.3	24.1
More than 5 months ago	3.7	2.9	4.8
**Why tennis training ceased**			
Not allowed access to a court	70.2	67.1	74.5
Not allowed to train with a partner	15.3	19.9	8.8
Don't want to take the risk	9.3	8.2	10.8
Other	5.2	4.8	5.9
**Off-court training location**			
Home	45.3	46.8	43.7
Local park	21.7	25.7	17.2
Sporting club	2.5	0.6	4.6
Other	10.3	8.2	12.6
Not training	20.2	18.7	21.9
**Equipment access**			
None	24.4	24.3	24.6
Bands/tubes	22.6	26.2	18.1
Cardio machines	7.4	6.3	8.8
Cones/ladders/hurdles	3.2	5.8	0.0
Free weights	26.8	26.7	26.9
Resistant machines	5.0	3.9	6.4
Other	10.6	6.8	15.2
**Why no off-court training is being conducted during COVID-19**	**(*****n*** **= 74)**	**(*****n*** **= 37)**	**(*****n*** **= 37)**
Unsure how to train during COVID-19	12.2	11.1	9.1
No equipment or space to train	35.1	26.7	31.8
Don't think off-court training is necessary	28.4	17.8	29.6
Lacking motivation to train	44.6	44.4	29.6

### Emotional Well-Being

**Figure 2:**
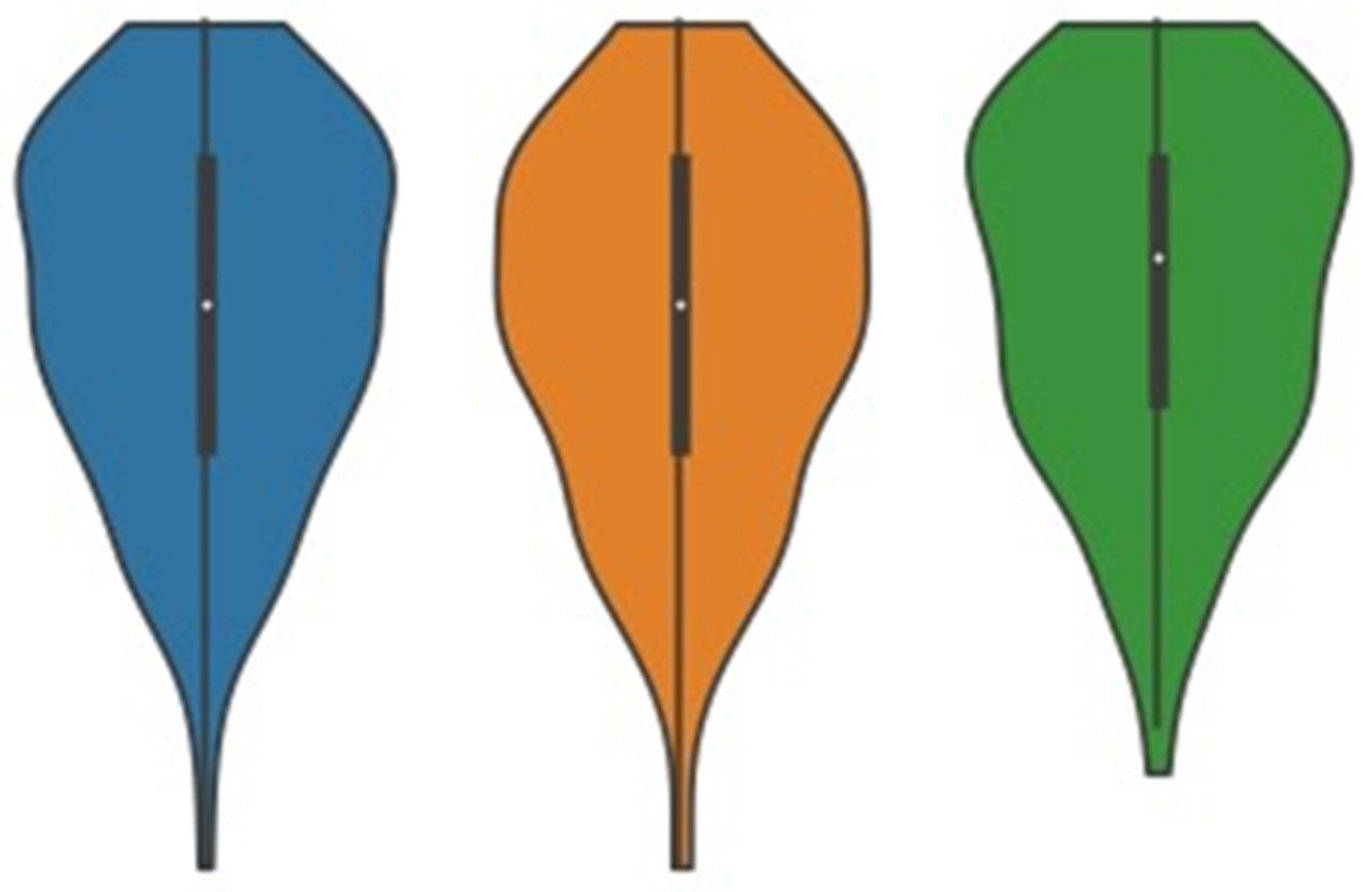
Mood state of all participants (blue) and those between the ages of 41 and 60 years (orange) and 61 years and over (green), using the Brief Emotional Experience Scale. A significant difference (*p* = 0.002) was found between the two age groups. The median for each group is indicated by the white dot, with the interquartile ranges shown by the thick black line. The remaining distribution, excluding outliers, are shown by the thin black line. Scores greater than 0 indicate a positive mood state, with scores below 0 indicating a negative mood state.

The BEES average score was 0.99 ± 1.27, indicating that respondents had a somewhat positive emotional well-being (greater than 0) during the COVID-19 pandemic (Figure 2). Senior participants had significantly (*P* = 0.002) greater values on the BEES (more positive) compared to middle-aged participants.

Of the selected variables, including tennis training, match play, age group, gender, and employment status only age group (*r* = 0.167, *p* = 0.002) and employment status (*r* = −0.129 *p* = 0.010) were significantly associated with the BEES score. However, after running corrections, there was no significant association between employment status and the BEES.

There was no statistical difference in emotional well-being between participants who participated in singles, doubles or both singles and doubles (*p* = 0.679). Additionally, the emotional well-being did not significantly differ depending on tennis experience (*p* = 0.184). Finally, the time period since tennis training cessation did not significantly influence the emotional well-being (*p* = 0.573) of the participants who had ceased tennis training.

## Discussion

Emerging literature shows that COVID-19 has had a significant and negative impact on the mental health of Australians (Newby et al., 2019). These findings are, however, non-specific in nature. No studies to date have investigated the impact of COVID-19 on community athletes, particularly with respect to training and match play and emotional well-being. Here, we investigated the impact of COVID-19 on the training status, match play and emotional well-being of community level tennis players. We hypothesized that community tennis players would have a reduction in training hours and number of matches played during the COVID-19 pandemic and this would be associated with lower emotional well-being scores.

Our findings showed a reduction in hours spent training tennis, strength, speed/agility and coordination per week in Australian tennis players during the COVID-19 pandemic. Interestingly, no differences between the middle aged and senior age groups were observed for any of the training modalities. These findings align with previous research reporting a reduction of training load and intensity (75% of respondents) in South African elite and semi-elite athletes across 15 sports (Pillay et al., 2020). Consistent with our expectations, the number of tennis matches played per week was dramatically reduced (88%) during the COVID-19 pandemic. Participants attributed this reduction in match play to restricted access to tennis courts. However, it is noteworthy that the number of matches played prior to and during the COVID-19 pandemic did not significantly differ between the two age groups. Contrary to our assumption, hours training endurance and flexibility/mobility per week did not significantly decrease during the COVID-19 pandemic. This may be due to the limited equipment and facilities needed to successfully undertake these training modalities. This explanation is supported by the finding that 24.4% of participants didn't have access to any exercise equipment during the COVID-19 pandemic. Whilst training modalities such as endurance and flexibility/mobility are sufficient to maintain physical health during the pandemic, the skill demands of tennis require sport-specific training and/or tennis matches to be regularly performed (Fernandez-Fernandez et al., 2017).

Existing studies have noted poor mental health outcomes, including greater emotional distress and concerns in younger adults during the COVID-19 pandemic (Ebrahimi et al., 2020; Huang and Zhao, 2020; Mazza et al., 2020; Qiu et al., 2020; Wang et al., 2020). These studies have consisted of newly established assessments, including the COVID-19 Peritraumatic Distress Index as well as previously established assessments, including the Depression, Anxiety and Stress Scale, Generalized Anxiety Disorder 7 and Health Anxiety Inventory. Though, it is important to note only two studies from China and Norway, respectively, have included a population group over the age of 60 years (Fernandez-Fernandez et al., 2017; Mazza et al., 2020). The results from our study indicate that although the emotional well-being of all participants was positive (score of ~1 on a scale ranging from −3 to +3), the senior group reported significantly higher (*p* = 0.002) scores in the BEES compared to the middle-aged group. This is in agreement with previous findings from Norway with associations found between higher age and lesser anxiety levels and greater adherence to mitigation strategies (Fernandez-Fernandez et al., 2017). Whist in our results the training hours and tennis matches played per week did not significantly differ between the age groups, one difference and possible explanation for variations in emotional well-being is the employment status of individuals prior to the COVID-19 pandemic. A significantly greater number of participants were employed on a full-time basis in the middle aged compared to the senior age group prior to the COVID-19 pandemic. It is speculated that the decrease in full-time employment found in the senior group may be attributed to participant retirement. Therefore, it is plausible that the financial impact of the COVID-19 pandemic was not as severe in the senior compared to the middle-aged group, which may explain their significantly more positive emotional well-being.

We expected that reduced training and match play would be associated with lower emotional well-being. Contrary to our expectations, we did not observe any meaningful associations between training hours or match play and emotional well-being. This finding was unexpected given the well-established literature noting a positive impact of physical activities, such as tennis, on mental health (Benedetti et al., 2008; Battaglia et al., 2016; Hekmati Pour and Hojjati, 2016). The lack of association between these outcomes may be explained by the short duration of the restrictions experienced in Australia at the time of the survey, however, this requires further examination in countries with restrictions over longer durations. Given that we did not examine emotional well-being scores prior to the pandemic, it is not known whether reduced match play and training would have contributed to its decline. Further studies are needed to explore whether COVID-19-related changes in training hours or match play mediate changes in emotional well-being.

While we did not observe significant associations between training and match play and emotional well-being outcomes, we did observe a significant association between employment status and emotional well-being outcomes. However, this weak association did not survive corrections. Furthermore, respondents who had ceased tennis training at various points in time as well as respondents of different tennis experience and match play formats showed no differences in emotional well-being. These findings may be a result of the sample consisting of solely community level tennis players, therefore, the influence of tennis training and match play as well as the other tennis-related variables may not be considerable. Given these findings it is expected other factors (e.g., physical and mental health or lifestyle) had a greater contribution to emotional well-being in Australian tennis players.

A number of limitations need to be considered when interpreting the findings of the present study. Firstly, the cross-sectional study design limits our ability to determine the causes of individuals emotional well-being scores. Secondly, the geographical location within Australia was not collected, therefore, it not known if the sample was biased by a particular state. Thirdly, the emotional well-being of individuals prior to COVID-19 is unknown and may have been influenced by factors outside of the pandemic (e.g., family background, finances, and health diseases). Fourthly, emotional well-being using as measured by the BEES is only one component of mental health. Finally, several survey questions required participants to respond retrospectively, this along with the self-selected responses of individuals may result in increased variation of scores. However, to ensure the anonymity of participants this was unavoidable.

Despite the aforementioned limitations, this is the first study to assess the impact of COVID-19 on training hours, matches played, and emotional well-being for community tennis players over the age of 40 years in Australia. The results from this study indicate the training hours and matches played per week significantly declined for community tennis players as a result of the COVID-19 pandemic. While not expected, the emotional well-being of community tennis players in Australia was not negatively affected through the COVID-19 pandemic, with the senior age group reporting significantly greater emotional well-being scores compared to the middle-aged group. The results of this study shed light on the impact COVID-19 and the subsequent restrictions have had on community tennis players.

## Data Availability Statement

The raw data supporting the conclusions of this article will be made available by the authors, without undue reservation.

## Ethics Statement

The studies involving human participants were reviewed and approved by Human Research Ethics Committee, Edith Cowan University. The patients/participants provided their written informed consent to participate in this study.

## Author Contributions

MT designed the study, collected, analyzed and interpreted the data, and drafted the manuscript. PB collected, analyzed and interpreted the data, and edited the manuscript. KN analyzed the data and edited the manuscript. OG analyzed the data and edited the manuscript. TC designed the study, collected, analyzed and interpreted the data, and edited the manuscript. All authors gave final approval on the accepted manuscript.

## Conflict of Interest

The authors declare that the research was conducted in the absence of any commercial or financial relationships that could be construed as a potential conflict of interest.
