# Supplementary material for: Influence of the COVID-19 Pandemic on Mood and Training in Australian Community Tennis Players
Source: Front Sports Act Living. 2021 Mar 18;3:589617. doi: 10.3389/fspor.2021.589617 (PMC8012798; doi:10.3389/fspor.2021.589617)
Supplement: Supplementary file 1 [file Data_Sheet_1.docx]

COVID-19 Tennis Players Survey

How do you describe yourself?

- Male
- Female
- Transgender
- Do not identify as female, male or transgender

My age:

▼

My relationship status:

- I am single
- I am presently in a committed relationship
- Other

My employment status prior to COVID-19:


*Note: This question also refers to self-employment. So for example if you work for yourself on full-time basis you would select 'full-time'.

▼

Have you lost income due to the COVID-19 situation?

- Yes
- No

What match format do you predominately play?

▼

How long have you been playing tennis?

▼

On average, prior to the COVID-19 situation how many hours a week were you training:

|  |  |
| --- | --- |
| Tennis | ▼ |
| Strength | ▼ |
| Endurance | ▼ |
| Speed & Agility | ▼ |
| Flexibility & Mobility | ▼ |
| Coordination (e.g. balance) | ▼ |
| Mental / tactics | ▼ |

| 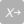 |
| --- |

On average, since the COVID-19 situation how many hours a week are you training:

|  |  |
| --- | --- |
| Tennis | ▼ |
| Strength | ▼ |
| Endurance | ▼ |
| Speed & Agility | ▼ |
| Flexibility & Mobility | ▼ |
| Coordination (e.g. balance) | ▼ |
| Mental / tactics | ▼ |

Display This Question:

If On average, since the COVID-19 situation how many hours a week are you training: = Tennis [ 0 ]

When did you cease tennis training?

▼

Display This Question:

If On average, since the COVID-19 situation how many hours a week are you training: = Tennis [ 0 ]

Why did you cease tennis training?

- I am not allowed to access a tennis court due to COVID-19 restrictions
- I am not allowed to train with my tennis partner/coach in person due to COVID-19 restrictions
- I don't want to take the risk of being infected by COVID-19
- Other (please specify) ________________________________________________

Display This Question:

If On average, since the COVID-19 situation how many hours a week are you training: = Strength [ 0 ]

And On average, since the COVID-19 situation how many hours a week are you training: = Endurance [ 0 ]

And On average, since the COVID-19 situation how many hours a week are you training: = Speed & Agility [ 0 ]

And On average, since the COVID-19 situation how many hours a week are you training: = Flexibility & Mobility [ 0 ]

And On average, since the COVID-19 situation how many hours a week are you training: = Mental / tactics [ 0 ]

What are the reasons for not performing off court training?
(e.g. strength, endurance or mental training)

- I don't think it's necessary to perform off court training during the COVID-19 situation
- I am unsure how to train during the COVID-19 situation
- I have no equipment/space for training during the COVID-19 situation
- I am lacking motivation to train during the COVID-19 situation

What equipment can you access for your physical training?

- None
- Free weights
- Bands/tubes
- Cones/ladders/hurdles
- Resistance Machines (e.g. leg press)
- Cardio machines (e.g. treadmill)
- Others (Please specify) ________________________________________________

Where are you performing your physical training?

- I'm not performing any physical training during the COVID-19 situation
- Home
- Local park
- Sporting club
- Others (Please specify) ________________________________________________

On average, prior to the COVID-19 situation how many matches were you playing per week?
(e.g. tournaments, league matches and training matches)

▼

On average, since the COVID-19 situation how many matches are you playing per week?
(e.g. tournaments, league matches and training matches)

▼

Have you contracted COVID-19?

- Yes
- Unsure
- No

I think the response of my tennis organisation to the COVID-19 outbreak has been:

▼

Over the past month I have generally been feeling emotionally:

|  | Not at all | A little bit | Quite a bit | A lot | Extremely |
| --- | --- | --- | --- | --- | --- |
| Happy |  |  |  |  |  |
| Worried |  |  |  |  |  |
| Calm |  |  |  |  |  |
| Sad |  |  |  |  |  |
| Confident |  |  |  |  |  |
| Afraid |  |  |  |  |  |
